# Pre-Treatment Fear of Weight Gain Is Associated With Engagement in a Greater Degree of Pre-Treatment Maladaptive Exercise Among Individuals With Binge-Spectrum Eating Disorders

**DOI:** 10.1002/erv.70090

**Published:** 2026-02-06

**Authors:** Naomi G. Hill, Elizabeth W. Lampe, Adrienne Juarascio, Stephanie M. Manasse

**Affiliations:** 1 Department of Psychology, Ohio University, Athens, Ohio, USA; 2 Department of Psychological and Brain Sciences, Drexel University, Philadelphia, Pennsylvania, USA; 3 Center for Weight Eating and Lifestyle Sciences, Drexel University, Philadelphia, Pennsylvania, USA; 4 Geisel School of Medicine, Dartmouth College, Lebanon, New Hampshire, USA; 5 Oregon Research Institute, Springfield, Oregon, USA; 6 Center for Healthcare Delivery Science, Nemours Children’s Health, Wilmington, Delaware, USA

**Keywords:** binge eating disorder, bulimia nervosa, cognitive behaviour therapy, exercise, fear of weight gain

## Abstract

**Objective:**

Individuals with binge-spectrum eating disorders (EDs) engage in varying degrees of maladaptive and adaptive exercise. Elevated shape/weight concern is associated with engagement in maladaptive *and* adaptive exercise. No research has examined whether *specific* facets of shape/weight concern (e.g., fear of weight gain) are associated with degree of maladaptive versus adaptive exercise engagement.

**Method:**

Participants were 124 adults with binge-spectrum EDs enroled in outpatient trials of Enhanced Cognitive Behaviour Therapy. Linear regression models examined associations between each facet of shape and weight concern concurrently at pre-treatment and degree of maladaptive versus adaptive exercise at pre-treatment (i.e., percentage maladaptive exercise episodes of total exercise episodes). We explored these relationships across treatment and diagnostic groups.

**Results:**

Greater pre-treatment fear of weight gain was associated with a greater degree of pre-treatment maladaptive exercise (*p* = 0.027). This pattern was marginally significant in the longitudinal model (*p* = 0.057) and was upheld within the BN-spectrum (*p*’s < 0.041) but not the BED-spectrum group.

**Discussion:**

Accounting for all other facets, fear of weight gain may function as a risk factor for engagement in a greater degree of maladaptive exercise pre- and post-treatment. Future research should examine the mechanisms underlying associations between fear of weight gain and maladaptive exercise engagement.

## 1 | Introduction

Individuals with eating disorders (EDs), including bulimia nervosa (BN) and binge-eating disorder (BED) engage in both maladaptive and adaptive exercise (Lampe et al. 2021). Maladaptive exercise, defined as any exercise that is compulsive or compensatory and intended to influence one’s body shape and weight (Fairburn et al. 2014; Meyer et al. 2011), is associated with poorer treatment outcomes (Bardone-Cone et al. 2016; Carter et al. 2004; Kostrzewa et al. 2013). Adaptive exercise is conceptualised as neither compulsive nor compensatory, may be performed for reasons such as socialising with others, improving one’s mood, or for enjoyment (Cash et al. 1994; Lampe et al. 2021), and is associated with lower eating pathology and improved treatment outcomes (Calogero and Pedrotty 2004; Lampe et al. 2022a, 2023a; Mathisen 2018; Mathisen et al. 2021; Mathisen et al. 2023; Pendleton et al. 2002). Prior research has largely characterised individuals with EDs as either maladaptive or adaptive exercisers (Gorrell et al. 2021), which limits understanding of risk factors for individuals who may engage in varying degrees of *both* types of exercise and prevents individualised treatment development. To improve theoretical models and intervention development, more research is needed to understand whether distinct risk factors are associated with a greater degree of engagement in maladaptive versus adaptive exercise.

### 1.1 | Shape and Weight Concern Is an Important Driver of ED Behaviours, Including Exercise

One important, but under-investigated risk-factor in relation to exercise is elevated shape and weight concern. Shape and weight concern comprises several unique facets including overvaluation of shape/weight, preoccupation with shape/weight, dissatisfaction with shape/weight, fear of weight gain, feeling fat, desire for a flat stomach, desire to lose weight, and negative reactions to prescribed weighing (Table 1; Fairburn et al. 2014; Fairburn and Beglin 1994). These facets show differential cross-sectional and prospective associations with several ED behaviours including fasting and purging (Askew et al. 2020; Sharpe et al. 2018). However, no studies to date have empirically examined whether, and to what degree, each facet of shape and weight concern is differentially associated with maladaptive or adaptive exercise engagement. For example, dissatisfaction with one’s shape and weight may increase the likelihood that an individual feels compelled to exercise. On the other hand, dissatisfaction with one’s body shape and weight contributes to *adaptive* exercise engagement within the context of healthy lifestyle interventions (Onu et al. 2024). Understanding differential associations between facets of shape and weight concern and degree of engagement in maladaptive versus adaptive exercise will inform the development of treatments to reduce maladaptive exercise and foster engagement in adaptive exercise.

### 1.2 | Facets of Shape and Weight Concern Are Differentially Associated With Maladaptive Exercise

Prior literature indicates that individual facets of shape and weight concern are associated with maladaptive exercise engagement among individuals with EDs. Among outpatient samples of individuals with EDs including BN and BED, elevations in overvaluation of shape and weight and fear of weight gain were predictive of future engagement in maladaptive exercise over periods of 1 week (Tabri et al. 2015), several hours (Lampe, Presseller, et al. 2023b), and across Enhanced Cognitive Behaviour Therapy for EDs (CBT-E; Srivastava et al. 2023). Fear of weight gain at age 14 also predicted maladaptive exercise engagement at age 24 in a large cohort study of adolescents and young adults (Schaumberg et al. 2023). Higher levels of body dissatisfaction were also associated with greater maladaptive exercise engagement cross-sectionally (Palermo and Rancourt 2023) and longitudinally (H. A. Davis et al. 2024) among university students. Taken together, overvaluation of shape and weight, fear of weight gain, and body dissatisfaction are associated with maladaptive exercise engagement across diagnoses. To our knowledge, no research has examined the *concurrent* contributions of specific facets of shape/weight concern to the degree of maladaptive exercise engagement, thus limiting our understanding of which facets may be the most relevant to target in treatment to reduce maladaptive exercise.

### 1.3 | Preliminary Evidence for the Association of Several Facets of Shape and Weight Concern and Adaptive Exercise

Interestingly, there is also evidence that several specific facets of shape and weight concern are associated with adaptive exercise engagement among individuals with EDs in studies that utilised ecological momentary assessment designs which deliver surveys to participants smartphones several times per day. One study found that momentary dissatisfaction with one’s body shape/weight was associated with a greater likelihood of engagement in both maladaptive *and* adaptive exercise at the next survey for individuals with binge-spectrum EDs (Srivastava et al. 2022). Another study found that increased momentary fear of weight gain was associated with a greater likelihood of engagement in *adaptive* compared to maladaptive exercise at the next survey in this same sample (Lampe, Wons, et al. 2022b). These studies both hypothesised that the observed links between facets of shape and weight concern and adaptive exercise engagement may be a result of momentary elevations in shape and weight concern captured at time 1 dissipating by the time adaptive exercise engagement was reported at time 2 (Lampe, Wons, et al. 2022b). Further investigating the perhaps paradoxical relationship between shape/weight concern and adaptive exercise engagement cross-sectionally and over a longer time period (e.g., across treatment) is critical for augmenting theoretical models of exercise in the context of EDs.

### 1.4 | Limitations of Existing Literature

Existing literature is limited by its examination of exercise engagement as either maladaptive-only or adaptive-only (Gorrell et al. 2021). Up to 43% of individuals with binge-spectrum EDs engage in *both* maladaptive and adaptive exercise (Lampe et al. 2021), yet there is no research evaluating specific risk factors for this significant subset of patients. To address limitations of the existing literature, the current study will leverage a continuous exercise outcome variable that represents the percent of exercise episodes that were maladaptive out of total exercise episodes. Higher percentages indicate a greater degree of maladaptive exercise engagement and lower percentages indicate a greater degree of adaptive exercise engagement. Understanding whether specific facets of shape and weight concern are associated with a greater degree of engagement in maladaptive versus adaptive exercise will augment theoretical models of exercise within EDs by moving beyond a binary classification scheme for exercise engagement and improve treatment development by increasing our understanding of which facets may be the most relevant to target in treatment to reduce maladaptive exercise.

Furthermore, no literature has examined the impact of treatment on these associations, limiting our understanding of mechanisms through which current interventions are functioning. Elevated pre-treatment shape and weight concern is associated with worse overall treatment outcome (Vall and Wade 2015). Understanding how *specific* facets of pre-treatment shape and weight concern impact exercise treatment outcomes may aid in the development of interventions to target specific pre-treatment elevations in shape and weight concern to reduce degree of engagement in maladaptive exercise and improve overall outcomes. More research is needed to better understand which facets of shape and weight concern are associated with degree of engagement in maladaptive versus adaptive exercise pre- and post- ED treatment so that patient outcomes can be improved by finding ways to decrease maladaptive exercise without decreasing adaptive exercise.

### 1.5 | The Current Study

The present study examined the extent to which each facet of shape and weight concern was associated with whether an individual engaged in a greater degree of maladaptive versus adaptive exercise pre- and post- CBT-E. We first examined cross-sectional associations between eight facets of shape and weight concern and exercise engagement. Based on prior research examining associations between facets of shape and weight concern with other compensatory behaviours (Askew et al. 2020; Sharpe et al. 2018), we hypothesised that pre-treatment preoccupation with shape and weight and over-valuation of shape and weight would be associated with a greater degree of maladaptive exercise type at pre-treatment. We explored longitudinal associations between the eight facets of shape and weight concern at pre-treatment and degree of maladaptive versus adaptive exercise engagement at post-treatment. Finally, given that elevated shape and weight concern are not a diagnostic criterion for BED as they are for BN (American Psychiatric Association 2013), we explored pre- and post-treatment associations between the eight facets of shape and weight concern and degree of maladaptive versus adaptive exercise engagement separately among individuals with BN- and BED-spectrum EDs.

## 2 | Methods

### 2.1 | Participants

The current data originate from pre-treatment and post-treatment evaluations of 124 individuals diagnosed with binge-spectrum EDs who participated in one of four clinical trials investigating enhanced cognitive behavioural therapy for binge eating (CBT-E) (Juarascio et al. 2022; Juarascio, Felonis, et al. 2021; Juarascio, Srivastava, et al. 2021; Manasse et al. 2020). Recruitment for parent studies occurred both locally in Philadelphia metropolitan area for face-to-face treatment studies and nationally for remote treatment studies. Eligibility criteria across all studies required participants to have reported at least one objective binge episode weekly over the past 3 months, be over 18 years old, and have a body mass index (BMI) above 17.5. Exclusion criteria across all parent studies included severe psychological conditions (such as significant substance dependence or active psychotic disorder), severe medical conditions precluding safe participation, diagnosis of anorexia nervosa (AN), acute suicide risk, concurrent treatment for EDs or weight loss, prior exposure to CBT-E, inability to communicate in English, or current or planned pregnancy or nursing. Participants included in the current analysis reported a minimum of 12 objective binge episodes and at least one exercise episode over the past month at pre- and post-treatment, given that the aim of the current study was to examine whether specific facets of shape/weight concern were associated with degree of engagement in maladaptive versus adaptive exercise. The presence/absence of features of maladaptive exercise (e.g., compulsive, compensatory exercise) were the outcome of interest; therefore, if participants were not engaging in any exercise, we were unable to observe anything about characteristics of that exercise. Detailed information regarding each parent study and specific inclusion/exclusion criteria is available in Supporting Information S1: Table S1.

### 2.2 | Treatment

All parent studies provided the same base CBT-E treatment, which consisted of weekly 50-min treatment sessions delivered face-to-face either in-person or over HIPAA compliant Zoom software. The CBT-E sessions covered self-monitoring, regular eating patterns, reducing dietary restraint, engaging in alternative activities, and addressing overvaluation of shape and weight. All research procedures including treatment and assessment received approval from the Drexel University Institutional Review Board.

### 2.3 | Measures

All individuals were administered the Eating Disorder Examination 17.0D (EDE; Fairburn et al. 2014) at pre- and post-treatment by a trained interviewer to evaluate exercise habits and shape and weight concern. The EDE is a thoroughly validated semi-structured interview designed to assess ED symptoms and behaviours. Eating disorder diagnosis (BN- vs. BED-spectrum) was also established based on reported behaviours during the EDE interview. Individuals were categorised as BN- spectrum if they reported ≥ 6 compensatory behaviours, and as BED-spectrum if they reported < 6 compensatory behaviours over the past 3 months at pre-treatment.

#### *EDE assessment of exercise*.

Consistent with past work (Lampe, Hill, et al. 2023a), in addition to standard questions in the EDE addressing compensatory exercise: ‘Over the past 4 weeks have you exercised as a means of controlling your weight, altering your shape or amount of fat, or burning off calories?’ compulsive exercise was identified using a modified EDE item ‘When you have exercised over the past 4 weeks, have you felt driven or compelled to exercise?’ Participants initially provided examples of exercises they had performed (e.g., walking, yoga, weight training) and the total frequency of all exercise sessions over the past month. Frequency of compensatory and compulsive exercise was assessed via the two items above, and a sum score was created from these items to capture all maladaptive exercise episodes. The frequency of adaptive exercise was determined by subtracting instances of maladaptive exercise from the total exercise frequency. Pre- and post-treatment exercise variables were computed by assigning participants a value corresponding to their proportion of maladaptive exercise episodes out of their total exercise episodes (e.g., a participant with 1 maladaptive episode and 19 adaptive episodes would be assigned a value of 5.00 and a participant with 19 maladaptive episodes and 1 adaptive episode would be assigned a value of 95.00).

#### *EDE assessment of shape & weight concern*.

EDE items assessing overvaluation of weight and shape (a composite score of two items from the shape concern and weight concern subscales; Cronbach’s *α* = 0.79), preoccupation with shape and weight, dissatisfaction with shape and weight (Cronbach’s *α* = 0.78), fear of weight gain, feeling fat, desire for flat stomach, desire to lose weight, and reactions to prescribed weighing were used to assess facets of shape and weight concerns. All facets of shape and weight concerns were assessed over the past 4 weeks and are coded from 0 (no endorsement of the facet over the past 4 weeks) to six (endorsement of the facet every day over the past 4 weeks or the highest degree of intensity over the past 4 weeks). To minimise collinearity, two facets of shape and weight concern (discomfort seeing one’s body and discomfort with exposing one’s body) were not included due to high correlation (*r* > 0.70) with other facets.

### 2.4 | Statistical Analysis

Although our specific aims and hypotheses of the study remain unchanged, we significantly altered our data analytic plan following the peer review process. Original analyses were pre-registered (https://osf.io/e297q/) and are reported in the Supporting Information S1 for transparency (see Supporting Information S1: Tables S2 and S3). All analyses were conducted in R version 4.3.2 (R Core Team 2023), and *α* was set at 0.05 across all models tested. We did not adjust for family-wise error rate given that we only ran one main model, included numerous covariates to increase robustness of our findings, and the overarching nature of the study was exploratory. The data met assumptions of linear regression. Identified outliers were retained in the current analyses as they appeared to be valid observations (e.g., one participant reported 28 exercise episodes in the past month, which was determined to be possible). All models covaried for parent study enrolment, ED diagnostic group (BN- or BED-spectrum), age, sex, and BMI. Standardised estimates are reported for all models.

#### Main Aim:

##### Cross-sectional associations between pre-treatment facets of shape and weight concern and pre-treatment exercise engagement.

A linear regression model examined associations between each facet of shape and weight concern concurrently and degree of maladaptive versus adaptive exercise at pre-treatment.

#### Exploratory Aim 1:

##### Longitudinal associations between pre-treatment facets of shape and weight concern and post-treatment exercise engagement.

A linear regression model examined associations between each facet of shape and weight concern at pre-treatment concurrently and degree of maladaptive versus adaptive exercise over the past month at post-treatment while adjusting for pre-treatment degree of maladaptive versus adaptive exercise engagement.

#### Exploratory Aim 2:

##### Associations between pre-treatment facets of shape and weight concerns and pre- and post-treatment exercise engagement by diagnostic group.

Linear regression models examined associations described above separately across diagnostic groups.

## 3 | Results

### 3.1 | Participant Demographics and Missing Data

The total sample included 124 participants ranging in age from 18 to 68 years old (*M* = 38.4, SD = 12.1). The parent studies (*n* = 155 in total) included participants who did not report engaging in any form of exercise at baseline (*n* = 31; 20.0%) and post-treatment (*n* = 40; 25.8%); these participants were excluded from analyses in the current study. Most participants identified as female (*n* = 106, 85.5%), followed by male (*n* = 18, 14.5%). Most participants identified as White (*n* = 96, 77.4%), followed by Black/African American (*n* = 12, 9.6%), Asian (*n* = 8, 6.4%), Other (*n* = 4, 3.2%), More than one race (*n* = 2, 1.6%), and Unknown/prefer not to say (*n* = 2, 1.6%). Nine participants (7.6%) identified as Hispanic/Latinx. The average BMI for the full sample was 31.1 kg/m^2^ (SD = 7.8). There were 84 participants with BN-spectrum EDs and 40 participants with BED-spectrum EDs at pre-treatment. There were 75 participants with BN-spectrum EDs and 40 participants with BED-spectrum EDs at post-treatment due to 9 participants in the BN-spectrum group not engaging in any exercise at post-treatment. Participant demographic characteristics by parent trial are reported in Supporting Information S1: Table S4.

In the full dataset of the four parent studies (*n* = 155 in total), 15.5% (*n* = 23) of the post-treatment exercise observations were missing as these participants did not provide any post-treatment data at all. Data were assumed missing at random as pre-treatment exercise type was not associated with post-treatment missingness. Consistent with our pre-registration, we handled missing data using multiple imputation with the mice package (Van Buuren et al. 2019). All predictors and covariates were included in imputation; only participant ID (which was never missing) and baseline/post-treatment exercise engagement were excluded from the imputation model. Models estimated five imputations, and all imputed data was checked for impossible values (e.g., shape and weight concern scores < 0 or > 6). We also inspected kernel density plots, looked for non-overlapping residual distributions, and compared observed and imputed data distributions to identify deviations in imputed values from the observed data. R code for imputation can be found in Supporting Information S1.

### 3.2 | Preliminary Analyses: Bivariate Correlations Between Facets of Shape and Weight Concern and Degree of Maladaptive Exercise Engagement

Zero order correlations between eight shape and weight concern facets and degree of maladaptive exercise engagement can be found in Table 2. Most facets of shape and weight concern exhibited moderate positive correlations with each other and degree of maladaptive exercise engagement exhibited the strongest correlation with fear of weight gain (*r* = 0.428, *p* < 0.001).

### 3.3 | Main Aim: Associations Between Pre-Treatment Facets of Shape and Weight Concern and Pre-Treatment Exercise Engagement

Over and above other facets of shape/weight concern and covariates, only greater pre-treatment fear of weight gain was associated with a greater degree of pre-treatment maladaptive exercise (*B* = 0.20, *p* = 0.027; Table 3).

### 3.4 | Exploratory Aim 1: Associations Between Pre-Treatment Facets of Shape and Weight Concern and Post-Treatment Exercise Engagement

The pattern of results held in the longitudinal model but the statistical significance did not. Over and above other facets of shape/weight concern, covariates, and pre-treatment exercise engagement, greater pre-treatment fear of weight gain was only marginally associated with a greater degree of post-treatment maladaptive exercise (*B* = 0.23, *p* = 0.057; Table 3).

### 3.5 | Exploratory Aim 2: Associations Between Pre-Treatment Facets of Shape and Weight Concern and Pre- and Post-Treatment Exercise Engagement Among Individuals With BN- Versus BED-Spectrum EDs

Among the sample with BN-spectrum EDs, pre-treatment fear of weight gain was associated with a greater degree of pre- (*B* = 0.30, *p* = 0.010) and post-treatment (*B* = 0.31, *p* = 0.040) maladaptive exercise over and above other facets of shape/weight concern and covariates (see Supporting Information S1: Table S5). None of the shape/weight concern facets were significantly associated with pre- or post-treatment exercise engagement in the BED-spectrum group (all *p*’s > 0.138; Supporting Information S1: Table S6).

## 4 | Discussion

The current study investigated the degree to which facets of shape and weight concern were associated with degree of engagement in maladaptive versus adaptive exercise pre- and post- CBT-E among a sample of 124 adults with binge-spectrum EDs. Greater pre-treatment fear of weight gain was associated with a greater degree of pre-treatment maladaptive exercise engagement. The pattern of results was attenuated in the longitudinal model for the full sample and held in the cross-sectional and longitudinal models in the BN-spectrum, but not BED-spectrum, group. Results converge with findings from the ecological momentary assessment literature (Lampe, Presseller, et al. 2023b), suggesting that fear of weight gain may function as both a between-person risk factor and within-person maintenance factor for engagement in a greater degree of maladaptive exercise pre- and post-CBT-E.

### 4.1 | Importance of Fear of Weight Gain in Relation to Degree of Maladaptive Exercise Engagement at Pre- and Post-Treatment

The current study was the first, to our knowledge, to examine contributions of each facet of shape and weight concern to exercise engagement *concurrently* and utilise a continuous, rather than categorical, outcome variable to represent degree of maladaptive, relative to adaptive, exercise engagement pre- and post-treatment. The positive bivariate correlation between fear of weight gain and degree of maladaptive exercise engagement was the strongest among all the facets, supporting our main findings that fear of weight gain emerged as the only significant cross-sectional and longitudinal predictor of degree of maladaptive exercise engagement. To our knowledge, no prior cross-sectional research has examined associations between fear of weight gain and engagement in maladaptive or adaptive exercise. Our longitudinal results, though marginally significant, map onto findings from a large cohort study in which fear of weight gain in adolescence predicted greater odds of engaging in maladaptive exercise in young adulthood (Schaumberg et al. 2023). Our results also align with past research in a combined clinical ED and university student sample which found that a composite measure of central ED fears (e.g., fear of disliking how one’s body feels due to weight gain) predicted engagement in maladaptive exercise over a 2-month period while accounting for levels of baseline maladaptive exercise engagement (Levinson and Williams 2020).

The current longitudinal findings add to the mixed literature on momentary associations between fear of weight gain and exercise, such that some findings indicate associations with maladaptive exercise engagement (Lampe, Presseller, et al. 2023b) whereas others have found associations with *adaptive* exercise engagement (Lampe, Wons, et al. 2022b). Converging findings in the current longitudinal study and prior momentary literature (Lampe, Presseller, et al. 2023b) give preliminary support for fear of weight gain as both a between-person risk factor and within-person maintenance factor for engagement in maladaptive exercise. Regarding conflicting findings with momentary literature (Lampe, Wons, et al. 2022b), perhaps it is true that momentary elevations in fear of weight gain captured at time 1 dissipate by the time adaptive exercise engagement is reported at time 2 *and* elevated fear of weight gain over the past 3 months predicts greater engagement in maladaptive exercise over the same time period. Future research should continue to disentangle how the timescale of associations between fear of weight and exercise engagement may impact the overall picture of this relationship.

Although the cross-sectional relationship between fear of weight gain and degree of engagement in maladaptive exercise was attenuated in the longitudinal model, the longitudinal relationship remained significant when tested in the BN-spectrum sub-sample. Prior literature indicates that overvaluation of shape and weight, another distinct facet of shape and weight concern, is reported more consistently by individuals with BN-versus BED-spectrum EDs (Grilo et al. 2009). Perhaps heterogeneity of shape and weight concerns with the BED-spectrum sample attenuated findings that were otherwise present in the BN-spectrum group. Although our sample was representative of those with binge-spectrum eating disorders, our sample size for the BED-spectrum diagnostic group was relatively small (*N* = 40) and underpowered to detect small to medium effect sizes. Future research should examine these relationships in larger samples of individuals with BED to better understand if relationships between fear of weight gain and degree of maladaptive exercise engagement differ by diagnosis.

The finding that fear of weight gain outperformed other facets in its relationship with maladaptive exercise engagement mirrors the network analysis literature which consistently indicates that fear of weight gain is a central ED symptom, particularly among participants with BN (Levinson et al. 2017; Sala et al. 2024). Links between fear of weight gain and maladaptive exercise are less consistent in the network analysis literature. Network analyses in treatment-seeking samples have not found strong connections between fear of weight gain and maladaptive exercise in the presence of other symptoms, including other facets of shape/weight concern (Forrest et al. 2018; Levinson et al. 2017; Meier et al. 2020). Future research should aim to replicate the findings from the current study in other samples where maladaptive exercise may be more central, such as those with individuals with longer illness duration (Christian et al. 2021) non-treatment-seeking samples (Cusack et al. 2021).

Findings from the current study also align well with theoretical models that conceptualise EDs within anxiety disorder (Schaumberg et al. 2021) and obsessive-compulsive disorder frameworks (C. Davis and Kaptein 2006; Meyer et al. 2011). Within anxiety disorders, engaging in a safety behaviour (e.g., only attending social gatherings with a close friend) temporarily reduces anxiety about the feared outcome (e.g., social rejection) but ultimately maintains the fear in the long run (Telch and Lancaster 2012). Similarly, in obsessive-compulsive disorder, compulsions (e.g., frequent hand washing) function to reduce frequency and intensity of obsessions (e.g., ‘I’m contaminated’) in the short-term, but fail to do so in the long-term. Maladaptive exercise may be conceptualised as a safety behaviour or compulsion that temporarily reduces anxiety about gaining weight, but ultimately maintains the fear or obsession (e.g., ‘I’m going to gain weight’) in the long run. Because fear of weight gain is currently inadequately targeted by CBT-E (Butler et al. 2023), conceptualising maladaptive exercise through an anxiety or obsessive-compulsive disorder lens may have direct implications for improving treatments. Schaumberg et al. (2021) recommend exposure-based treatments as a viable path forward for reducing fear of weight gain and its consequences (e.g., maladaptive exercise) among individuals with EDs. Violating a patient’s expectancies about occurrence of a feared outcome is a particularly powerful component of exposure-based treatments for anxiety-related disorders (Craske et al. 2014). Eating disorder clinicians may mirror this aspect of exposure-based treatments by asking patients to predict how much weight they will gain by refraining from engagement in maladaptive exercise or reducing their frequency or intensity of exercise engagement during a given week of treatment. If patients find that their expectations of weight gain are violated by this exposure, the link between maladaptive exercise and fear of weight gain may be weakened. Future research should test these ideas in exposure-based interventions for maladaptive exercise.

### 4.2. | Strengths and Limitations

The current study’s primary strength was the examination of a group of participants who engage in varying degrees of maladaptive and adaptive exercise to better capture the heterogeneity of exercise engagement among individuals with EDs (Lampe et al. 2021). We utilised a continuous, rather than categorical definition of exercise engagement (Gorrell et al. 2021) to be inclusive of individuals who engage in *both* maladaptive and adaptive exercise. We also leveraged a well-validated semi-structured diagnostic interview to establish ED diagnoses, frequency of shape/weight concern, and exercise engagement, utilised a sample of patients with binge-spectrum EDs to increase generalisability of results, and adjusted for relevant covariates of age, sex, BMI, diagnosis, and parent study enrolment in all models.

However, our findings should be interpreted in the context of limitations. First, over 80% of our participants identified as female; future research should examine these associations in samples with a sufficient number of males to examine sex as a moderator as there is evidence that shape and weight concern and exercise engagement within EDs may differ between sexes (Lavender et al. 2017). Second, while the current study was the first to utilise a continuous exercise outcome, percentage-based metrics obscure information about raw number of exercise episodes. Future research should continue to explore alternative ways of conceptualising adaptive and maladaptive exercise continuously. Third, although the longitudinal nature of our data is a strength, we were unable to examine how these relationships functioned at long-term follow-up after treatment. Future research may consider examining whether and how long fear of weight gain persists following CBT-E and whether post-treatment changes in fear of weight gain may continue to drive engagement in maladaptive exercise. Relatedly, given the observational nature of the study, we were unable to determine whether pre-treatment fear of weight gain *causes* maladaptive exercise engagement at post-treatment. Fourth, due to lack of availability of audio recordings for EDE interviews, we were unable to double code interviews for calculating estimates of inter-rater reliability. While this represents a limitation given that individual rater differences may impact EDE rating, Drexel University employs a robust EDE training process including didactic training, mock interviews, and shadowing of assessments. All EDE raters across the four trials attended a weekly consensus meeting for the duration of the studies and were expected to reach 80% agreement with trained raters who had over 100 h of experience on several EDE interviews before completing interviews independently. We acknowledge the lack of inter-rater reliability as a limitation given that the impact of an individual rater may influence outcomes. Finally, whereas we were able to capture the continuous nature of exercise engagement, adaptive exercise episodes were defined as any episode of exercise that was *not* classified as maladaptive. The absence of maladaptive features of exercise (e.g., feeling driven or compelled, exercising to burn off calories) may not necessarily imply that an exercise episode is adaptive. It will be important for future research to better define what constitutes an adaptive exercise episode and whether various adaptive reasons for exercise (e.g., for socialising, for health benefits) are differentially associated with shape and weight concerns.

## 5. | Conclusions

The present study was the first to concurrently examine eight facets of shape and weight concern in relation to degree of engagement in maladaptive versus adaptive exercise pre- and post-treatment. Greater pre-treatment fear of weight gain was significantly associated with a greater degree of engagement in pre-treatment maladaptive exercise and marginally associated with a greater degree of engagement in post-treatment maladaptive exercise. These patterns were strengthened among individuals with BN-spectrum EDs, while they did not hold among individuals with BED-spectrum EDs. Future research should investigate the mechanisms by which fear of weight gain is cross-sectionally and longitudinally associated with maladaptive exercise engagement to improve treatment efficacy.

## Supplementary Material

2

Additional supporting information can be found online in the Supporting Information section.

Supporting Information S1: erv70090-sup-0001-suppl-data.docx.

## Figures and Tables

**TABLE 1 |:** Definitions and clinical examples of the 10 facets of shape and weight concerns measured by the eating disorder examination and eating disorder examination-questionnaire (Fairburn et al. 2014; Fairburn and Beglin 1994).

Shape/Weight concern facet	Definition	Clinical example
Desire to lose weight	A strong desire to lose weight; can be present even for individuals who are unaware of their weight.	“If I lose 10 pounds, I’ll be so much happier.”
Desire for a flat stomach	A definite desire to have a flat or concave stomach.	“I would look so much better with a flat stomach.”
Dissatisfaction with shape and weight	A general negative attitude towards one’s shape or weight because it is viewed as too large.	“I often feel upset that my body is too large. It never looks thin enough.”
Fear of weight gain	A definite fear of gaining weight or gaining more weight.	“I don’t know how I would live if I gained more weight, it’s all I think about.”
Feeling fat	A multidimensional construct that comprises emotional and physical sensations and is not fully explained by one’s objective body weight; distinct from feeling bloated around one’s menstrual cycle.	“I always feel like the fattest person in the room. I feel uncomfortable in my skin.”
Importance of shape and weight	Occurs when shape and weight are the primary criteria used to evaluate oneself as a person.	“Gaining weight makes me feel like a failure.
Preoccupation with shape and weight	Persistent thoughts about one’s body shape and weight that leads to trouble concentrating on or thinking about other things.	“When I’m having a conversation with a friend, I find myself losing track of what we are talking about because I can’t stop thinking about how my body looks.”
Reactions to prescribed weighing	The strength of negative reaction to the prospect of being weighed once per week during eating disorder treatment, patients engage in collaborative weekly weighing with their therapist.	“If I was only able to weigh myself once a week, I would be so upset. My whole day would be ruined.”

**TABLE 2 |:** Zero-order correlations and descriptive statistics among eight baseline facets of shape and weight concern and degree of maladaptive exercise engagement.

	1	2	3	4	5	6	7	8	9
1. Desire to lose weight	—								
2. Desire for a flat stomach	0.289**	—							
3. Dissatisfaction	0.670***	0.371***	—						
4. Fear of weight gain	0.518***	0.416***	0.499***	—					
5. Feeling fat	0.566***	0.285**	0.627***	0.390***	—				
6. Importance	0.408***	0.410***	0.480***	0.405***	0.233**	—			
7. Preoccupation	0.299***	0.286**	0.364***	0.368***	0.256**	0.244**	—		
8. Reaction to prescribed weighing	0.236**	0.308***	0.300***	0.249**	0.109	0.212*	0.061	—	
9. Degree of maladaptive exercise engagement	0.317***	0.395**	0.324***	0.428***	0.160	0.338***	0.196*	0.278**	—
Mean	4.52	2.78	4.45	3.36	4.89	4.35	1.60	2.70	0.53
SD	2.18	2.75	1.33	2.57	1.90	1.34	2.05	1.88	0.45
Range	0–6	0–6	0–6	0–6	0–6	0–6	0–6	0–6	0–1

* *p* < 0.05.

** *p* < 0.01.

*** *p* < 0.001.

**TABLE 3 |:** Cross-sectional and longitudinal associations between facets of weight and shape concern and type of exercise engagement.

Facet	*B* (SE)	95% CI	*p*
Cross-sectional associations			
Desire to lose weight	0.03 (0.10)	−0.17, 0.23	0.746
Desire for a flat stomach	0.07 (0.08)	−0.10, 0.24	0.430
Dissatisfaction with weight/shape	0.04 (0.11)	−0.18, 0.27	0.700
Fear of weight gain	0.20 (0.09)	0.02, 0.37	0.027*
Feeling fat	−0.01 (0.10)	−0.21, 0.18	0.907
Importance of weight/shape	0.07 (0.08)	−0.10, 0.23	0.430
Preoccupation with weight/shape	0.03 (0.08)	−0.13, 0.19	0.702
Reactions to prescribed weighing	0.04 (0.08)	−0.11, 0.19	0.614
Study 2^a^	−0.60 (0.19)	−0.99, −0.22	0.003**
Study 3^a^	−0.45 (0.33)	−1.10, 0.21	0.180
Study 4^a^	−0.50 (0.19)	−0.88, −0.13	0.009**
Age	−0.06 (0.07)	−0.20, 0.09	0.416
BMI	0.04 (0.08)	−0.10, 0.19	0.554
Sex^b^	−0.34 (0.20)	−0.74, 0.07	0.102
Diagnosis^c^	0.85 (0.18)	0.49, 1.20	< 0.001***
Longitudinal associations			
Desire to lose weight	−0.07 (0.14)	−0.35, 0.22	0.649
Desire for a flat stomach	0.04 (0.11)	−0.19, 0.26	0.746
Dissatisfaction with weight/shape	−0.15 (0.16)	−0.47, 0.17	0.358
Fear of weight gain	0.23 (0.12)	−0.01, 0.48	0.057
Feeling fat	0.10 (0.13)	−0.16, 0.36	0.447
Importance of weight/shape	−0.11 (0.11)	−0.34, 0.12	0.335
Preoccupation with weight/shape	−0.02 (0.11)	−0.24, 0.20	0.833
Reactions to prescribed weighing	0.05 (0.11)	−0.16, 0.27	0.620
Study 2^a^	−0.61 (0.30)	−1.21, −0.02	0.043*
Study 3^a^	0.25 (0.44)	−0.62, 1.12	0.572
Study 4^a^	−0.74 (0.29)	−1.32, −0.17	0.011*
Age	0.12 (0.11)	−0.09, 0.33	0.246
BMI	−0.10 (0.11)	−0.31, 0.12	0.370
Sex^b^	0.14 (0.29)	−0.43, 0.71	0.625
Diagnosis^c^	−0.26 (0.28)	−0.82, 0.30	0.352
Pre-treatment exercise	0.26 (0.13)	< 0.001, 0.52	0.049*

*Note:* Standardised estimates are reported.

a Study 1 is the reference group.

b Males are the reference group.

c BED-spectrum is the reference group.

* *p* < 0.05.

** *p* < 0.01.

*** *p* < 0.001.

## Data Availability

De-identified data from this study are not available in a public archive. De-identified data from this study will be made available (as allowable according to institutional IRB standards) by emailing the corresponding author. Analytic code used to conduct the analyses presented in this study are available by emailing the corresponding author. Materials used to conduct the study are available upon reasonable request to the corresponding author.

## Highlights

- Accounting for all other facets of shape/weight concern, greater pre-treatment fear of weight gain was associated with a greater degree of pre-treatment maladaptive exercise.
- This pattern of results held across treatment and among individuals in the bulimia nervosa-spectrum group, but not among individuals in the binge-eating-disorder spectrum group.
- Future research should examine the mechanisms underlying associations between fear of weight gain and maladaptive exercise engagement to improve treatments targets.
