# Supplementary material for: Pre-Treatment Fear of Weight Gain Is Associated With Engagement in a Greater Degree of Pre-Treatment Maladaptive Exercise Among Individuals With Binge-Spectrum Eating Disorders
Source: Eur Eat Disord Rev. Author manuscript; Available in PMC 2026 Apr 30. (PMC13126317; doi:10.1002/erv.70090)
Supplement: 2 [file NIHMS2163272-supplement-2.docx]

**Supplementary Table 1.** Parent study-specific information.

| **Clinical Trial** | **Study description** | **Number of sessions** | **Study specific inclusion criteria** | **Study specific exclusion criteria** |
| --- | --- | --- | --- | --- |
| **Project COMPASS**:  Juarascio, Felonis, et al., 2021 | Project COMPASS recruited 256individuals who were assigned to one of 16 treatment combinations to identify the optimal combination off our acceptance-based treatment components adjunctive to CBT-E(mindful awareness, distress tolerance, emotion modulation, values-based decision making). The current analyses included 7 individuals randomized to receive base CBT-E only with no acceptance-based components. | 16 | None | None |
| **The Acquire Project**:  Juarascio, Srivastava, et al., 2021 | The Acquire Project recruited 56 participants (n = 41 included in the current analyses) who completed electronic self-monitoring in a smartphone app and received just-in-time-adaptive interventions (JITAIs)or no JITAIs alongside 16 sessions of CBT-E. | 16 | - ≥12 compensatory behaviors in the past 3 months - Willingness to use smartphone application to track eating episodes during treatment | - History of bariatric surgery |
| **SenseSupport Study**:  Juarascio et al., 2022 | The SenseSupport Study recruited 30adults (n = 28 included in the current analyses) who received CBT-E augmented with JITAIs delivered via smartphone app addressing episodes of fasting >5 h detected by a continuous glucose monitor. | 12 | - ≥12 episodes of fasting for 5+waking hours in the last 4 weeks | - Not on stable psychiatric medication for at least 1 month - Diagnosis of diabetes - Taking medication known to impact insulin or glucose levels - History of bariatric surgery |
| **Project REBOOT**:  Manasse et al., 2020 | Project REBOOT recruited 63participants (n = 48 included in the current analyses) who received a12-week course of CBT-E for binge eating, while completing active inhibitory control trainings or control “sham” trainings daily for the first 4 weeks and weekly in weeks 5–12. | 12 | None | - High baseline inhibitory control - Binge episodes consists primarily of uncooked fruits and vegetables, as per a modified Food Frequency Questionnaire - Prescribed stimulant medication |

**Method and Results Consistent with the Pre-Registered Analytic Plan**

**Method**

**Measures**

*EDE Assessment of Exercise*

Consistent with past work (Lampe, Hill, et al., 2023), in addition to standard questions in the EDE addressing compensatory exercise: “Over the past four weeks have you exercised as a means of controlling your weight, altering your shape or amount of fat, or burning off calories?” compulsive exercise was identified using a modified EDE item “When you have exercised over the past 4 weeks, have you felt driven or compelled to exercise?” Participants initially provided examples of exercises they had performed (e.g., walking, yoga, weight training) and the total frequency of all exercise sessions over the past month. Frequency of compensatory and compulsive exercise was assessed via the two items above, and a sum score was created from these items to capture all maladaptive exercise episodes. The frequency of adaptive exercise was determined by subtracting instances of maladaptive exercise from the total exercise frequency. Pre-treatment exercise type was categorized as follows: 1 = adaptive exercise only, 2 = both maladaptive and adaptive exercise reported, 3 = maladaptive exercise only.

**Statistical Analysis**

All analyses were pre-registered (<https://osf.io/e297q/>) and conducted in R version 4.3.2 (R Core Team, 2023), and α was set at 0.05 across all models tested. The assumption of a linear relationship between the predictor (facets of shape and weight concern) and the logit transformation of the outcome variable (exercise types) was violated in our data. As such, we included quadratic terms for each predictor variable in the models to account for non-linear relationships. Statistically significant (i.e., *p*<.05) higher-order terms are interpreted. Identified outliers were retained in the current analyses as they appeared to be valid observations (e.g., a participant reported 28 exercise episodes in the past month, which was determined to be possible). All models covaried for parent study enrollment, ED diagnostic group (BN- or BED-spectrum), age, sex, and BMI.

***Main Aim:*** *Associations between pre-treatment facets of shape and weight concern and* pre-treatment *exercise engagement*. Multinomial logistic regression models examined to what extent each facet of shape and weight concern predicted whether an individual reported maladaptive-only, both maladaptive and adaptive, or adaptive-only exercise at pre-treatment.

***Exploratory Aim 1:*** *Associations between pre-treatment facets of shape and weight concern and post-treatment exercise engagement.* Multinomial logistic regression models examined to what extent each facet of shape and weight concern at pre-treatment predicted whether an individual reported maladaptive-only, both maladaptive and adaptive, or adaptive-only exercise over the past month at post-treatment.

In addition to the aims above, we pre-registered that we would model cross-sectional and longitudinal associations between facets of shape and weight concern and pre- and post-treatment exercise engagement among individuals with BN-spectrum and BED-spectrum EDs separately. However, small cell sizes in the BED-spectrum group (see Supplementary Table 2) and ceiling effects on the shape/weight concern predictor variables in the BN-spectrum group, led to problems with convergence in our models. As such, we were unable to complete this pre-registered exploratory analysis.

**Results**

**Main Aim: Associations between pre-treatment facets of shape and weight concern and pre-treatment type of exercise engagement**

Those with greater desire to lose weight at pre-treatment were more likely to engage in maladaptive-only exercise at pre-treatment compared to adaptive-only exercise (OR=1.61; *p*=.006; Supplementary Table 2) *and* both maladaptive and adaptive exercise (OR=1.71, *p*=.002; Supplementary Figure 1a). Until a rating of “4” on the EDE (i.e., having a definite desire to lose weight on 16-22 of the past 28 days), participants were most likely to engage in *both* maladaptive and adaptive exercise, followed by adaptive-only, then maladaptive-only. As ratings on the EDE approached “5” (i.e., having a definite desire to lose weight on 23-27 of the past 28 days), this pattern inverted, such that these participants were most likely to engage in maladaptive-only exercise, followed by adaptive-only and then both maladaptive and adaptive exercise.

Those with greater dissatisfaction with shape and weight at pre-treatment were more likely to engage in adaptive-only exercise at pre-treatment compared to both maladaptive and adaptive exercise (OR=.62, *p*=.034; Supplementary Figure 1b). Until a rating of “3” on the EDE (i.e., experiencing dissatisfaction with one’s body shape and weight on 13-15 of the past 28 days), participants were most likely to engage in both maladaptive and adaptive exercise, followed by maladaptive-only, then adaptive-only exercise. As ratings approached “5” on the EDE (i.e., experiencing dissatisfaction with one’s body shape and weight on 23-27 of the past 28 days), this pattern inverted, such that participants were most likely to engage in adaptive-only exercise, followed by maladaptive-only and both maladaptive and adaptive exercise.

**Exploratory Aim 1: Associations between pre-treatment facets of shape and weight concern and post-treatment type of exercise engagement**

The cross-sectional associations did not hold in the longitudinal model (Supplementary Table 3). However, those with greater fear of weight gain at pre-treatment were more likely to engage in maladaptive-only exercise at post-treatment compared to both maladaptive and adaptive exercise (OR=6.37, *p*=.044; Supplementary Figure 1c). At a rating of “0” on fear of weight gain on the EDE (i.e., experiencing fear of gaining weight on 0 of the past 28 days), participants were most likely to engage in adaptive-only exercise at post-treatment, followed by both maladaptive and adaptive exercise and maladaptive-only exercise. However, as ratings on the EDE approached “5” (i.e., experiencing fear of gaining weight on 23-27 of the past 28 days) on the EDE, this pattern inverted, such that these participants were most likely to engage in maladaptive-only exercise, followed by both maladaptive and adaptive and adaptive-only exercise.

**Supplementary Figure 1.** Quadratic relationships between facets of shape and weight concern and exercise type.


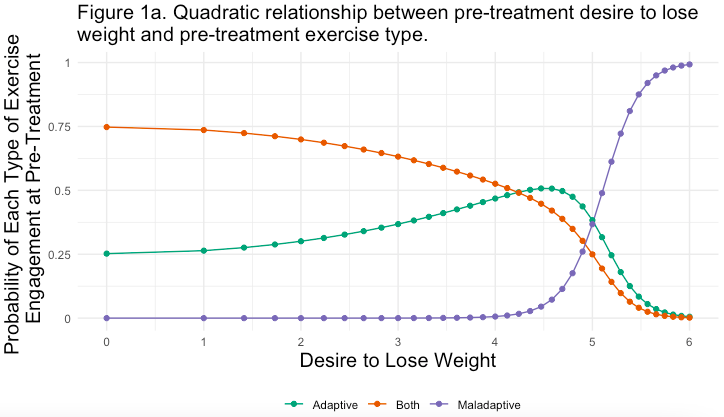


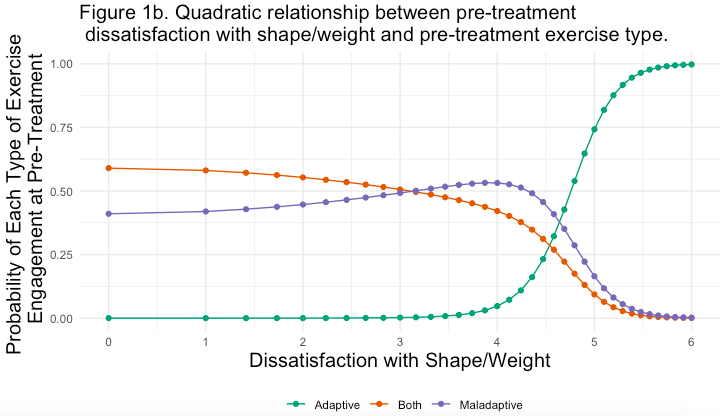


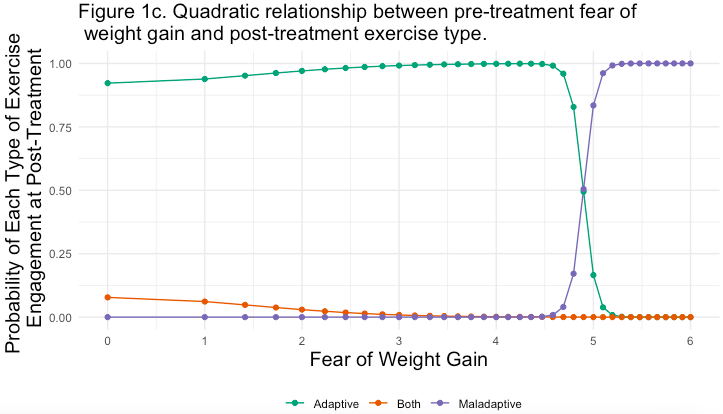


**Supplementary Table 2.** Cross-sectional associations between facets of weight and shape concern and type of exercise engagement.

| **Facet** | **Comparison** | **Term** | **Estimate (SE)** | **Odds Ratio** | **95% CI** | ***p*** |
| --- | --- | --- | --- | --- | --- | --- |
| Desire to lose weight | Maladaptive v. Adaptive | Linear | -3.37 (1.23) | .03 | .003, .38 | .006** |
|  |  | Quadratic | .48 (.17) | 1.61 | 1.15, 2.26 | .006** |
|  | Both v. Adaptive | Linear | .67 (.89) | 1.95 | .34, 11.20 | .456 |
|  |  | Quadratic | -.06 (.12) | .94 | .74, 1.20 | .624 |
|  | Maladaptive v. Both | Linear | -4.04 | .02 | .001, .22 | .422 |
|  |  | Quadratic | .54 | 1.71 | 1.22, 2.41 | .002* |
| Desire for a flat stomach | Maladaptive v. Adaptive | Linear | .75 (.87) | 2.12 | .38, 11.74 | .388 |
|  |  | Quadratic | -.10 (.14) | .91 | .69, 1.19 | .490 |
|  | Both v. Adaptive | Linear | .55 (.84) | 1.73 | .33, 9.07 | .514 |
|  |  | Quadratic | -.09 (.14) | .91 | .69, 1.19 | .497 |
|  | Maladaptive v. Both | Linear | .20 | 1.22 | .31, 4.91 | .775 |
|  |  | Quadratic | -.002 | 1.00 | .80, 1.24 | .984 |
| Dissatisfaction with weight/shape | Maladaptive v. Adaptive | Linear | 3.44 (1.63) | 31.11 | 1.28, 753.3 | .035* |
|  |  | Quadratic | -.44 (.22) | .65 | .42, 1.00 | .050 |
|  | Both v. Adaptive | Linear | 2.83 (1.60) | 16.92 | .74, 386.2 | .076 |
|  |  | Quadratic | -.47 (.22) | .62 | .40, .96 | .034* |
|  | Maladaptive v. Both | Linear | .61 | 1.84 | .07, 45.65 | .710 |
|  |  | Quadratic | .04 | 1.04 | .67, 1.60 | .866 |
| Fear of weight gain | Maladaptive v. Adaptive | Linear | .03 (.81) | 1.03 | .21, 4.99 | .973 |
|  |  | Quadratic | .06 (.13) | 1.06 | .38, 1.36 | .644 |
|  | Both v. Adaptive | Linear | -.37 (.61) | .69 | .21, 2.28 | .547 |
|  |  | Quadratic | .05 (.10) | 1.05 | .87, 1.28 | .604 |
|  | Maladaptive v. Both | Linear | .39 | 1.48 | .32, 6.79 | .613 |
|  |  | Quadratic | .01 | 1.01 | .80, 1.27 | .957 |
| Feeling fat | Maladaptive v. Adaptive | Linear | .26 (1.21) | 1.30 | .12, 13.95 | .832 |
|  |  | Quadratic | -.07 (.16) | .94 | .68, 1.29 | .685 |
|  | Both v. Adaptive | Linear | -1.33 (1.04) | .27 | .03, 2.04 | .202 |
|  |  | Quadratic | .13 (.14) | 1.14 | .86, 1.51 | .367 |
|  | Maladaptive v. Both | Linear | 1.58 | 4.87 | .45, 52.23 | .191 |
|  |  | Quadratic | -.20 | .82 | .60, 1.12 | .217 |
| Importance of weight/shape | Maladaptive v. Adaptive | Linear | -.29 (2.02) | .75 | .01, 39.01 | .885 |
|  |  | Quadratic | .11 (.25) | 1.11 | .69, 1.80 | .664 |
|  | Both v. Adaptive | Linear | 1.20 (1.40) | 3.32 | .21, 51.88 | .392 |
|  |  | Quadratic | -.04 (.18) | .96 | .68, 1.37 | .841 |
|  | Maladaptive v. Both | Linear | -1.49 | .22 | .003, 15.75 | .491 |
|  |  | Quadratic | .14 | 1.15 | .70, 1.90 | .577 |
| Preoccupation with weight/shape | Maladaptive v. Adaptive | Linear | 1.67 (.83) | 5.29 | 1.05, 26.78 | .044* |
|  |  | Quadratic | -.24 (.14) | .79 | .60, 1.03 | .085 |
|  | Both v. Adaptive | Linear | 1.39 (.78) | 4.02 | .87, 18.68 | .076 |
|  |  | Quadratic | -.17 (.13) | .85 | .65, 1.09 | .200 |
|  | Maladaptive v. Both | Linear | .27 | 1.32 | .35, 4.97 | .686 |
|  |  | Quadratic | -.07 | .93 | .75, 1.16 | .531 |
| Reactions to prescribed weighing | Maladaptive v. Adaptive | Linear | .69 (.79) | 1.99 | .42, 9.38 | .386 |
|  |  | Quadratic | -.09 (.15) | .92 | .68, 1.24 | .570 |
|  | Both v. Adaptive | Linear | 1.10 (.75) | 3.00 | .69, 12.98 | .141 |
|  |  | Quadratic | -.15 (.14) | .86 | .66, 6.26 | .288 |
|  | Maladaptive v. Both | Linear | -.41 | .66 | .13, 3.36 | .619 |
|  |  | Quadratic | .06 | 1.06 | .79, 1.44 | .695 |
| Study 2 ^a^ | Maladaptive v. Adaptive | | -3.50 (1.05) | .03 | .002, .35 | .005** |
|  | Both v. Adaptive | | -.22 (1.26) | .80 | .10, 6.26 | .832 |
|  | Maladaptive v. Both | | -3.28 | .04 | .003, .34 | .003** |
| Study 3 ^a^ | Maladaptive v. Adaptive | | -.74 (1.89) | .48 | .01, 23.1 | .709 |
|  | Both v. Adaptive | | 2.47 (1.98) | 11.88 | .29, 484.5 | .191 |
|  | Maladaptive v. Both | | -3.21 | .04 | .002, .78 | .034* |
| Study 4 ^a^ | Maladaptive v. Adaptive | | -2.67 (1.03) | .07 | .01, .54 | .010* |
|  | Both v. Adaptive | | -1.44 (1.05) | .24 | .03, 1.80 | .163 |
|  | Maladaptive v. Both | | -1.23 | .29 | .04, 2.02 | .212 |
| Age | Maladaptive v. Adaptive | | .003 (.03) | 1.00 | .93, 1.08 | .941 |
|  | Both v. Adaptive | | .05 (.04) | 1.06 | .99, 1.13 | .114 |
|  | Maladaptive v. Both | | -.05 | .95 | .89, 1.01 | .111 |
| BMI | Maladaptive v. Adaptive | | .07 (.05) | 1.07 | .96, 1.20 | .220 |
|  | Both v. Adaptive | | .06 (.06) | 1.06 | .97, 1.16 | .167 |
|  | Maladaptive v. Both | | .01 | 1.01 | .91, 1.12 | .875 |
| Sex ^b^ | Maladaptive v. Adaptive | | -.98 (1.36) | .38 | .03, 4.40 | .435 |
|  | Both v. Adaptive | | 2.03 (1.26) | 7.64 | .53, 110.5 | .136 |
|  | Maladaptive v. Both | | -3.01 | .05 | .003, .74 | .030* |
| BN v. BED Spectrum ^c^ | Maladaptive v. Adaptive | | 4.01 (.83) | 54.89 | 5.72, 526.8 | .001** |
|  | Both v. Adaptive | | .95 (1.15) | 2.60 | .51, 13.25 | .251 |
|  | Maladaptive v. Both | | 3.05 | 21.13 | 2.03, 219.6 | .011* |

*Note.*

^a^ Study 1 is the reference group.

^b^ Males are the reference group.

^c^ BED-spectrum is the reference group

**p*<.05

**p*<.01

****p*<.001

**Supplementary Table 3.** Longitudinal associations between facets of weight and shape concern and type of exercise engagement.

| **Facet** | **Comparison** | **Term** | **Estimate (SE)** | **Odds Ratio** | **95% CI** | ***p*** |
| --- | --- | --- | --- | --- | --- | --- |
| Desire to lose weight | Maladaptive v. Adaptive | Linear | 5.08 (3.64) | 161.2 | .13, >1,000 | .163 |
|  |  | Quadratic | -.84 (.53) | .43 | .15, 1.23 | .116 |
|  | Both v. Adaptive | Linear | .42 (2.11) | 1.52 | .02, 95.71 | .843 |
|  |  | Quadratic | -.01 (.26) | .99 | .59, 1.66 | .970 |
|  | Maladaptive v. Both | Linear | 4.68 (4.18) | 107.6 | .03, 3.90 | .263 |
|  |  | Quadratic | -.83 (.59) | .44 | .14, 1.39 | .161 |
| Desire for a flat stomach | Maladaptive v. Adaptive | Linear | 3.83 (2.15) | 46.22 | .68, 3,128 | .075 |
|  |  | Quadratic | -.51 (.31) | .60 | .33, 1.10 | .098 |
|  | Both v. Adaptive | Linear | 1.20 (.76) | 3.31 | .74, 14.79 | .117 |
|  |  | Quadratic | -.21 (.12) | .81 | .64, 1.02 | .077 |
|  | Maladaptive v. Both | Linear | 2.64 (2.20) | 14.00 | .19, 1,039 | .230 |
|  |  | Quadratic | -.31 (.32) | .74 | .40, 1.37 | .335 |
| Dissatisfaction with weight/shape | Maladaptive v. Adaptive | Linear | -2.21 (3.25) | .11 | <.01, 63.88 | .496 |
|  |  | Quadratic | .18 (.36) | 1.20 | .60, 2.40 | .611 |
|  | Both v. Adaptive | Linear | -2.12 (2.66) | .12 | <.01, 21.79 | .425 |
|  |  | Quadratic | .23 (.32) | 1.26 | .67, 2.36 | .482 |
|  | Maladaptive v. Both | Linear | -.11 (4.11) | .90 | <.01, 2,856 | .979 |
|  |  | Quadratic | -.05 (.47) | .96 | .38, 2.40 | .923 |
| Fear of weight gain | Maladaptive v. Adaptive | Linear | -9.15 (5.41) | <.01 | <.01, 4.24 | .091 |
|  |  | Quadratic | 1.60 (.90) | 4.94 | .84, 29.10 | .078 |
|  | Both v. Adaptive | Linear | 1.69 (1.05) | 5.42 | .69, 42.66 | .109 |
|  |  | Quadratic | -.25 (.16) | .78 | .56, 1.07 | .117 |
|  | Maladaptive v. Both | Linear | -10.84 (5.51) | <.01 | <.01, .96 | .049* |
|  |  | Quadratic | 1.85 (.92) | 6.37 | 1.05, 38.57 | .044* |
| Feeling fat | Maladaptive v. Adaptive | Linear | 7.23 (5.27) | 1,374 | .05, >1,000 | .170 |
|  |  | Quadratic | -1.02 (.72) | .36 | .09, 1.48 | .156 |
|  | Both v. Adaptive | Linear | -1.79 (1.65) | .17 | .01, 4.22 | .277 |
|  |  | Quadratic | .22 (.22) | 1.25 | .81, 1.92 | .312 |
|  | Maladaptive v. Both | Linear | 9.01 (5.38) | 8219 | .22, >1,000 | .094 |
|  |  | Quadratic | -1.24 (.73) | .29 | .07, 1.22 | .091 |
| Importance of weight/shape | Maladaptive v. Adaptive | Linear | 4.60 (3.67) | 99.69 | .08, >1,000 | .209 |
|  |  | Quadratic | -.80 (.52) | .45 | .16, 1.25 | .125 |
|  | Both v. Adaptive | Linear | -1.59 (2.41) | .20 | <.01, 22.83 | .509 |
|  |  | Quadratic | .15 (.29) | 1.16 | .66, 2.03 | .614 |
|  | Maladaptive v. Both | Linear | 6.19 (4.14) | 488.3 | .15, >1,000 | .135 |
|  |  | Quadratic | -.94 (.57) | .39 | .13, 1.18 | .095 |
| Preoccupation with weight/shape | Maladaptive v. Adaptive | Linear | 3.00 (2.23) | 20.13 | .25, 1.60 | .179 |
|  |  | Quadratic | -.52 (.41) | .60 | .27, 1.34 | .213 |
|  | Both v. Adaptive | Linear | .35 (.81) | 1.42 | .29, 6.92 | .668 |
|  |  | Quadratic | -.03 (.14) | .97 | .74, 1.29 | .853 |
|  | Maladaptive v. Both | Linear | 2.65 (2.33) | 14.22 | .15, 1,379 | .255 |
|  |  | Quadratic | -.49 (.43) | .61 | .26, 1.43 | .256 |
| Reactions to prescribed weighing | Maladaptive v. Adaptive | Linear | 1.46 (1.75) | 4.28 | .14, 131.7 | .405 |
|  |  | Quadratic | -.12 (31) | .89 | .48, 1.62 | .692 |
|  | Both v. Adaptive | Linear | -.13 (.88) | .88 | .16, 4.87 | .879 |
|  |  | Quadratic | .17 (.16) | 1.18 | .86, 1.63 | .306 |
|  | Maladaptive v. Both | Linear | 1.59 (1.91) | 4.89 | .12, 204.8 | .404 |
|  |  | Quadratic | -.29 (.33) | .75 | .39, 1.43 | .381 |
| Study 2 ^a^ | Maladaptive v. Adaptive | | -10.17 (4.77) | <.01 | <.01, .44 | .033* |
|  | Both v. Adaptive | | -.23 (1.01) | .80 | .11, 5.81 | .824 |
|  | Maladaptive v. Both | | -9.93 (4.80) | <.01 | <.01, .59 | .038* |
| Study 3 ^a^ | Maladaptive v. Adaptive | | -2.63 (3.45) | .07 | <.01, 62.25 | .446 |
|  | Both v. Adaptive | | -1.68 (1.83) | 5.34 | .15, 194.2 | .361 |
|  | Maladaptive v. Both | | -4.30 (3.82) | .01 | <.01, 24.44 | .261 |
| Study 4 ^a^ | Maladaptive v. Adaptive | | -15.50 (7.81) | <.01 | <.01, .82 | .047* |
|  | Both v. Adaptive | | -.85 (1.17) | .43 | .04, 4.25 | .470 |
|  | Maladaptive v. Both | | -14.66 (7.85) | <.01 | <.01, 2.07 | .062 |
| Age | Maladaptive v. Adaptive | | .07 (.05) | 1.07 | .96, 1.19 | .217 |
|  | Both v. Adaptive | | -.01 (.04) | .99 | .91, 1.07 | .750 |
|  | Maladaptive v. Both | | .08 (.06) | 1.08 | .96, 1.22 | .197 |
| BMI | Maladaptive v. Adaptive | | -.12 (.16) | .89 | .65, 1.21 | .449 |
|  | Both v. Adaptive | | -.07 (.07) | .94 | .81, 1.08 | .348 |
|  | Maladaptive v. Both | | -.05 (.17) | .95 | .68, 1.31 | .753 |
| Sex | Maladaptive v. Adaptive | | 1.96 (1.99) | 7.08 | .14, 349.8 | .325 |
|  | Both v. Adaptive | | -1.66 (1.25) | .19 | .02, 2.19 | .183 |
|  | Maladaptive v. Both | | 3.61 (2.17) | 37.15 | .53, 2.59 | .095 |
| BN v. BED Spectrum ^c^ | Maladaptive v. Adaptive | | -8.41 (4.15) | >1,000 | <.01, .76 | .043* |
|  | Both v. Adaptive | | 14.28 (3.58) | >1,000 | 1442, >10,000 | <.001*** |
|  | Maladaptive v. Both | | -19.28 (5.39) | <.001 | <.01, <.01 | <.001*** |

*Note.*

^a^ Study 1 is the reference group.

^b^ Males are the reference group.

^c^ BED-spectrum is the reference group

**p*<.05

***p*<.01

****p*<.001

**Supplementary Table 4.** Participant descriptives by study.

|  | **Study 1** | **Study 2** | **Study 3** | **Study 4** |  |
| --- | --- | --- | --- | --- | --- |
|  | **Project Acquire** | **SenseSupport** | **COMPASS** | **Project REBOOT** | **Full Sample** |
| Number of participants | 41 | 28 | 7 | 48 | 124 |
| Age: *M (SD)* | 40.0 (13.8) | 36.5  (11.3) | 30.9  (5.5) | 39.4  (11.5) | 38.4  (12.1) |
| Body mass index: *M (SD)* | 29.9 (7.0) | 30.7  (6.0) | 32.5  (10.0) | 32.2  (8.9) | 31.1  (7.8) |
| Female: *N (%)* | 34 (82.9) | 25  (89.3) | 5  (71.4) | 42  (87.5) | 106  (85.5) |
| Race: *N (%)* | | | | |  |
| White | 27 (65.9) | 24  (85.7) | 5  (71.4) | 40  (83.3) | 96  (77.4) |
| Black | 5 (12.2) | 2 (7.1) | 2 (28.6) | 3 (6.3) | 12 (9.6) |
| Asian | 4 (9.8) | 2 (7.1) | 0 (0) | 2 (4.2) | 8 (6.4) |
| American Indian/Alaska Native | 0 (0) | 0 (0) | 0 (0) | 0 (0) | 0 (0) |
| Hawaiian/Pacific Islander | 0 (0) | 0 (0) | 0 (0) | 0 (0) | 0 (0) |
| More than one | 0 (0) | 0 (0) | 0 (0) | 2 (4.2) | 2 (1.6) |
| Unknown/prefer not to say | 1 (2.4) | 0 (0) | 0 (0) | 1 (2.1) | 2 (1.6) |
| Other | 4 (9.8) | 0 (0) | 0 (0) | 0 (0) | 4 (3.2) |
| Hispanic/Latinx: *N (%)* | 2 (4.9) | 2 (7.1%) | 0 (0) | 5 (10.4) | 9 (7.6) |
| Exercise engagement at pre-treatment (days in past month): *M (SD)* | | | | |  |
| Adaptive exercise | 6.9 (1.1) | 7.4 (8.2) | 8.6 (9.7) | 8.1 (7.7) | 6.2 (8.0) |
| Maladaptive exercise | 9.9 (7.8) | 7.3 (8.9) | 6.1 (9.9) | 5.7 (8.5) | 7.5 (8.6) |

**Supplementary Table 5.** Cross-sectional and longitudinal associations between facets of weight and shape concern and type of exercise engagement among individuals with bulimia nervosa-spectrum disorders.

| **Facet** | **Estimate (SE)** | **95% CI** | ***p*** |
| --- | --- | --- | --- |
| **Cross-sectional associations** |  |  |  |
| Desire to lose weight | .03 (.15) | -.28, .33 | .851 |
| Desire for a flat stomach | .19 (.11) | -.03, .42 | .095 |
| Dissatisfaction with weight/shape | .21 (.15) | -.08, .51 | .158 |
| Fear of weight gain | .30 (.12) | .07, .53 | .010* |
| Feeling fat | -.07 (.15) | -.37, .23 | .642 |
| Importance of weight/shape | .07 (.11) | -.16, .30 | .530 |
| Preoccupation with weight/shape | -.07 (.11) | -.29, .16 | .564 |
| Reactions to prescribed weighing | .08 (.10) | -.12, .27 | .430 |
| Study 2 ^a^ | -.46 (.25) | -.95, .03 | .063 |
| Study 3 ^a^ | -.51 (.43) | -1.37, .36 | .247 |
| Study 4 ^a^ | -.27 (.25) | -.77, .23 | .293 |
| Age | -.17 (.10) | -.38, .04 | .103 |
| BMI | .13 (.11) | -.08, .34 | .214 |
| Sex ^b^ | -.55 (.34) | -1.22, .13 | .110 |
| **Longitudinal associations** |  |  |  |
| Desire to lose weight | -.01 (.20) | -.41, .38 | .940 |
| Desire for a flat stomach | .10 (.14) | -.18, .38 | .484 |
| Dissatisfaction with weight/shape | -.31 (.20) | -.71, .09 | .130 |
| Fear of weight gain | .31 (.15) | .01, .60 | .040* |
| Feeling fat | .12 (.18) | -.25, .48 | .522 |
| Importance of weight/shape | -.15 (.14) | -.44, .13 | .273 |
| Preoccupation with weight/shape | -.02 (.15) | -.32, .29 | .919 |
| Reactions to prescribed weighing | .06 (.13) | -.19, .32 | .613 |
| Study 2 ^a^ | -.38 (.33) | -1.04, .28 | .250 |
| Study 3 ^a^ | .40 (.51) | -.63, 1.42 | .441 |
| Study 4 ^a^ | -.54 (.32) | -1.19, .11 | .101 |
| Age | .14 (.14) | -.14, .42 | .310 |
| BMI | -.20 (.14) | -.48, .08 | .162 |
| Sex ^b^ | .22 (.46) | -.71, 1.14 | .638 |
| Pre-treatment exercise | .26 (.14) | -.03, .54 | .074 |

*Note.*

^a^ Study 1 is the reference group.

^b^ Males are the reference group.

**p*<.05

**p*<.01

****p*<.001

**Supplementary Table 6.** Cross-sectional and longitudinal associations between facets of weight and shape concern and type of exercise engagement among individuals with binge-eating-spectrum disorders.

| **Facet** | **Estimate (SE)** | **95% CI** | ***p*** |
| --- | --- | --- | --- |
| **Cross-sectional associations** |  |  |  |
| Desire to lose weight | .05 (.20) | -.35, .45 | .801 |
| Desire for a flat stomach | -.11 (.15) | -.43, .21 | .482 |
| Dissatisfaction with weight/shape | -.08 (.24) | -.58, .42 | .751 |
| Fear of weight gain | .04 (.20) | -.37, .45 | .839 |
| Feeling fat | -.25 (.21) | -.69, .19 | .248 |
| Importance of weight/shape | .15 (.17) | -.20, .51 | .384 |
| Preoccupation with weight/shape | .02 (.15) | -.29, .33 | .878 |
| Reactions to prescribed weighing | .02 (.17) | -.34, .38 | .918 |
| Study 2 ^a^ | -2.50 (.62) | -3.77, -1.23 | <.001*** |
| Study 3 ^a^ | -2.61 (.95) | -4.57, -.66 | .011* |
| Study 4 ^a^ | -2.82 (.55) | -3.95, -1.68 | <.001*** |
| Age | .12 (.16) | -.21, .44 | .465 |
| BMI | .06 (.15) | -.25, .37 | .691 |
| Sex ^b^ | -.12 (.32) | -.77, .54 | .711 |
| **Longitudinal associations** |  |  |  |
| Desire to lose weight | .17 (.23) | -.31, .65 | .468 |
| Desire for a flat stomach | -.30 (.20) | -.71, .11 | .138 |
| Dissatisfaction with weight/shape | .25 (.29) | -.36, .86 | .405 |
| Fear of weight gain | -.28 (.22) | -.75, .18 | .219 |
| Feeling fat | -.22 (.26) | -.77, .33 | .413 |
| Importance of weight/shape | .13 (.22) | -.34, .59 | .574 |
| Preoccupation with weight/shape | -.13 (.18) | -.50, .24 | .467 |
| Reactions to prescribed weighing | .27 (.21) | -.18, .71 | .224 |
| Study 2 ^a^ | -3.24 (1.19) | -5.72, -.76 | .013* |
| Study 3 ^a^ | -.86 (1.53) | -4.05, 2.33 | .579 |
| Study 4 ^a^ | -3.04 (1.19) | -5.54, -.55 | .019* |
| Age | .20 (.20) | -.21, .62 | .320 |
| BMI | .25 (.19) | -.15, .64 | .204 |
| Sex ^b^ | -.01 (.39) | -.83, .81 | .978 |
| Pre-treatment exercise | -.15 (.19) | -.54, .24 | .428 |

*Note.*

^a^ Study 1 is the reference group.

^b^ Males are the reference group.

^c^ BED-spectrum is the reference group

**p*<.05

**p*<.01

****p*<.001

**Multiple Imputation Code**

**Imputation code:**

# examine patterns of missingness

# rr: both variables are observed

# rm: the first variable is observed and the second one is missing

# mr: the first variable is missing and the second one is observed

# mm: both are missing

md.pattern(master)

md.pairs(master)

# 5 imputations of missingness excluding outcome, ID, and study variables

library(mice)

predictors <- quickpred(master, exc = c("Participant_IDNumber", “BL_AdEx”, “BL_MalEx”, “PT_AdEx”, “PT_MalEx”))

imp <- mice(data = master, predictorMatrix = predictors, m = 5, maxit = 1, seed = 32, print = FALSE)

#extract the imputed data sets from the imp object

data1.imp <- complete(imp)

data2.imp <- complete(imp,2)

data3.imp <- complete(imp,3)

data4.imp <- complete(imp,4)

data5.imp <- complete(imp,5)

# combine imputations for analysis

implist <- mids2mitml.list(imp)
